# Comparative analysis of mitochondrial genomes of geographic variants of the gypsy moth, *Lymantria dispar*, reveals a previously undescribed genotypic entity

**DOI:** 10.1038/s41598-017-14530-6

**Published:** 2017-10-27

**Authors:** Abdelmadjid Djoumad, Audrey Nisole, Reza Zahiri, Luca Freschi, Sandrine Picq, Dawn E. Gundersen-Rindal, Michael E. Sparks, Ken Dewar, Don Stewart, Halim Maaroufi, Roger C. Levesque, Richard C. Hamelin, Michel Cusson

**Affiliations:** 1 Laurentian Forestry Centre, Canadian Forest Service, Natural Resources Canada, 1055 rue du PEPS, Quebec City, Quebec, G1V 4C7 Canada; 2 Canadian Food Inspection Agency, Ottawa Plant Laboratory, Entomology Unit, Ottawa, Ontario, Canada; 3 Institute for Integrative and System Biology, 1030 Avenue de la Médecine, Université Laval, Quebec City, Quebec, G1V 0A6 Canada; 4 United States Department of Agriculture - ARS Invasive Insect Biocontrol and Behavior Laboratory, Beltsville, Maryland 20705 United States of America; 5 McGill University and Genome Quebec Innovation Centre, 740 Dr. Penfield Avenue Rm 7104, Montreal, Quebec, H3A 0G1 Canada; 6 University of British Columbia, Vancouver, British Columbia Canada

## Abstract

The gypsy moth, *Lymantria dispar* L., is one of the most destructive forest pests in the world. While the subspecies established in North America is the European gypsy moth (*L. dispar dispar*), whose females are flightless, the two Asian subspecies, *L. dispar asiatica* and *L. dispar japonica*, have flight-capable females, enhancing their invasiveness and warranting precautionary measures to prevent their permanent establishment in North America. Various molecular tools have been developed to help distinguish European from Asian subspecies, several of which are based on the mitochondrial barcode region. In an effort to identify additional informative markers, we undertook the sequencing and analysis of the mitogenomes of 10 geographic variants of *L. dispar*, including two or more variants of each subspecies, plus the closely related *L. umbrosa* as outgroup. Several regions of the gypsy moth mitogenomes displayed nucleotide substitutions with potential usefulness for the identification of subspecies and/or geographic origins. Interestingly, the mitogenome of one geographic variant displayed significant divergence relative to the remaining variants, raising questions about its taxonomic status. Phylogenetic analyses placed this population from northern Iran as basal to the *L. dispar* clades. The present findings will help improve diagnostic tests aimed at limiting risks of AGM invasions.

## Introduction

The gypsy moth, *Lymantria dispar* (Linnaeus, 1758) (Insecta: Lepidoptera: Erebidae: Lymantriinae), is considered one of the most destructive forest defoliators over much of its range. In North America alone, losses and population suppression operations targeting this species are estimated at $3.2 billion each year^1^. Currently, *L. dispar* comprises three recognized sub-species^2^ based on morphological criteria, female flight capability and geographic origins: *L. dispar dispar* Linnaeus, found over most of western Europe (“EGM” for European Gypsy Moth) and North Africa, and accidentally introduced from France into North America in 1869^3^, *L. dispar asiatica* Vnukovskij, distributed over much of continental Asia, including Russia, China and the Korean peninsula, and *L. dispar japonica* Motschulsky, which appears to be confined to Japan (Honshu, Shikoku, Kyushu and Hokkaido). For regulatory purposes, the latter two subspecies, along with three other closely related Japanese *Lymantria* species (i.e., *L. umbrosa* Butler, *L. postalba* Inoue and *L. albescens* Hori and Umeno), are generally considered “Asian gypsy moth” (AGM)^2^.

Unlike their European counterpart, the two Asian *L. dispar* subspecies have flight-capable females^4,5^ and a broader host range^6^, considerably increasing the risks of rapid propagation relative to EGM. With the current intensification of commercial trade with Asia, AGM introduction and establishment into North America are considered a very significant threat. AGM specimens are regularly intercepted at North American ports, and accidental introductions have occurred, resulting in costly eradication campaigns^7^.

The rigorous and rapid identification of intercepted gypsy moth samples suspected of being AGM and an assessment of their geographic origins are critical to the negotiations undertaken by North American regulatory agencies with their Asian trading partners in an effort to reduce risks of accidental introductions. However, morphological characters provide little useful information for species and subspecies delineation and for the identification of source countries, particularly when dealing with the immatures stages (e.g., eggs and larvae). As a consequence, many studies have examined the usefulness of molecular markers to help distinguish gypsy moth subspecies and to identify the geographic origins of intercepted specimens (see^8^). In this respect, a 658 bp fragment of the mitochondrial *cytochrome c oxidase subunit I* (COI) gene has received much attention, given its recognized effectiveness in delineating taxa^9^. However, although the COI 5’ barcode region could easily separate the European from the two Asian subspecies, it proved inadequate to distinguish *L. d. asiatica* from *L. d. japonica* ^8,10,11^. On the other hand, distinct regions of the mitochondrial genome, including the 3′ portion of the COI gene^8^ and five other genes^12^ (ND2, ND6, ATP6, ATP8 and CytB) provided resolution of the two Asian strains, pointing to the informativeness of alternative mitochondrial regions.

With respect to identifying the geographic origins of gypsy moth specimens, previous work has shown that the COI barcode alone could provide some clues as to the source of unknown samples^10,11^, and that other mitochondrial genes could also be useful for this purpose^12^. While analysis of nuclear markers such as microsatellites^12^ and genome-wide SNPs^13^ points to their remarkable value in identifying the geographic origins of gypsy moth specimens, mitochondrial genomes should not be underestimated as a potential source of markers to help identify geographic variants, especially in view of their high substitution rate relative to nuclear genomes^14,15^.

For the present study, we undertook the sequencing of the entire mitochondrial genome of 10 geographic variants of *Lymantria dispar*, with a good coverage of this species’ geographic range and inclusion of two or more variants of each subspecies; the closely related *L. umbrosa* was selected as outgroup. Only three *L. dispar* mitochondrial genomes had previously been sequenced and deposited in GenBank, each with limited information about the origin and subspecies designation of the insect from which they were obtained (accession numbers: FJ617240, GU994783, GU994784). Here, we present a detailed comparative analysis of gypsy moth mitochondrial genomes with the aim of assessing their usefulness for subspecies delimitation and identification of geographic origins. Interestingly, the mitochondrial genome of one of the geographic variants sampled in this study displayed important differences relative to the remaining variants. We thus conducted phylogenetic analyses to help shed light on the potential taxonomic position of this previously uncharacterized geographic variant.

## Results

### *Lymantria dispar* mitochondrial genome organization

**Figure 1:**
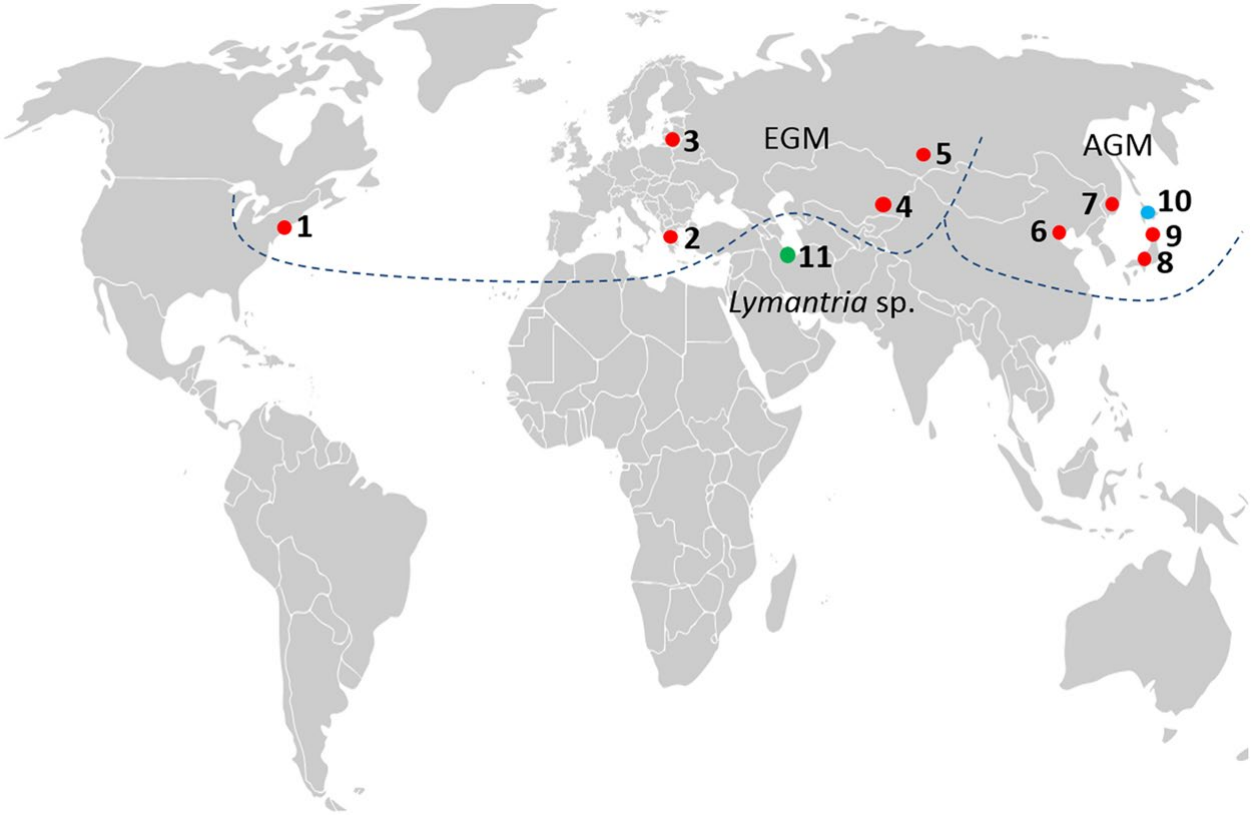
Sampling locations for *Lymantria* specimens used in this study. Red symbols represent sampling locations for *L. dispar* subspecies (**1**–**9**), blue symbol is for *L. umbrosa* (**10**) and green symbol is for the specimen from Iran (**11**). For full location names, see Table 1. Background map is a cropped version of the one available at: https://commons.wikimedia.org/wiki/Maps_of_the_world#/media/File:BlankMap-World-v2.png (for information about license, follow the same link).

A total of 11 mitochondrial genomes of *Lymantria* specimens (Table 1; Fig. 1) were completely sequenced, assembled and annotated (Table 2). While one of these specimens was *L. umbrosa*, the other 10 specimens were originally considered to be geographic variants of *L. dispar*. However, one sample from northern Iran (referred to here as L?_IR) displayed important differences relative to the other *L. dispar* mitogenomes, calling into question its taxonomic status (see details in the next section). To minimize the risk of misidentification, all specimens were assayed using a TaqMan qPCR method^8^ as well as cross-checked with their DNA barcodes in BOLD. At least two specimens identified here as *L. dispar dispar*, based on their mitochondrial genome sequences (Ldd_RB and Ldd_KZ; Table 1), are believed to be strongly admixted *dispar-asiatica* populations on the basis of their nuclear genotypes^8,12,13^.

**Table 1:** List of the *Lymantria* specimens processed for mitogenome sequencing.

	Map (Fig. 1)	Name	Species^1^	COI marker	Origin	Region	Supplier
EGM	1	Ldd_NJ	*L. dispar*	*dispar*	USA	New Jersey	D. Gundersen-R^3^
	2	Ldd_KG	*L. dispar*	*dispar*	Greece	Kavála, Macedonia	M. Keena^4^
	3	Ldd_LJ	*L. dispar*	*dispar*	Lithuania	Juodkrante, Kuzsin Nezijos	M. Keena^4^
	4	Ldd_KZ	*L. dispar*	*dispar*	Kazakhstan	Chuy Valley	S.K. Korb^5^
	5	Ldd_RB	*L. dispar*	*dispar*	Russia	Krasnoyarsk, Siberia	M. Keena^4^
AGM	6	Lda_TJ	*L. dispar*	*asiatica*	China	Tianjin	H. Nadel^6^
	7	Lda_RM	*L. dispar*	*asiatica*	Russia	Primorski, far east	M. Keena^4^
	8	Ldj_JN	*L. dispar*	*japonica*	Japan	Honshu	M. Keena^4^
	9	Ldj_ID	*L. dispar*	*japonica*	Japan	Iwate district	H. Nadel^6^
	10	Lu_JH	*L. umbrosa*	*umbrosa*	Japan	Hokkaido	C. Hideyuki^7^
	11	L?_IR^2^	*L. dispar?*	*dispar?*	Iran	Noor, Mazandaran	H. Rajaei^8^

^1^As determined using TaqMan assay^8^.

^2^Initially considered to be *L. dispar dispar* on the basis of TaqMan assay results^8^; results of full mitogenome analysis subsequently called this conclusion into question.

^3^USDA, Beltsville, Maryland, USA.

^4^US Forest Service, Hamden, CT, USA.

^5^Russian Entomological Society, Nizhny Novgorod, Russia.

^6^USDA APHIS, Buzzards Bay, MA, USA.

^7^Bernice Pauahi Bishop Museum, Honolulu, USA.

^8^State Museum of Natural History, Stuttgart, Germany.

**Table 2:** Sequenced *Lymantria* mitochondrial genomes: size, A + T content and accession numbers.

Species/subspecies*	Code	Size (bp)	A + T content (%)	Accession number
*L. dispar dispar*	Ldd_NJ	15,679	80.1	KY798442
*L. dispar dispar*	Ldd_KG	15,698	80.1	KY923062
*L. dispar dispar*	Ldd_LJ	15,688	80.2	KY923063
*L. dispar dispar*	Ldd_KZ	15,642	80.1	KY923065
*L. dispar dispar*	Ldd_RB	15,591	80.1	KY923064
*L dispar asiatica*	Lda_TJ	15,592	79.9	KY923067
*L dispar asiatica*	Lda_RM	15,593	79.9	KY923059
*L. dispar japonica*	Ldj_JN	15,605	80.0	KY923061
*L. dispar japonica*	Ldj_ID	15,616	80.0	KY923060
*L. umbrosa*	Lu_JH	15,642	80.0	KY923066
*L. dispar?*	L?_IR	15,651	80.2	KY923068

^*^As determined using TaqMan assay^8^.

**Figure 2:**
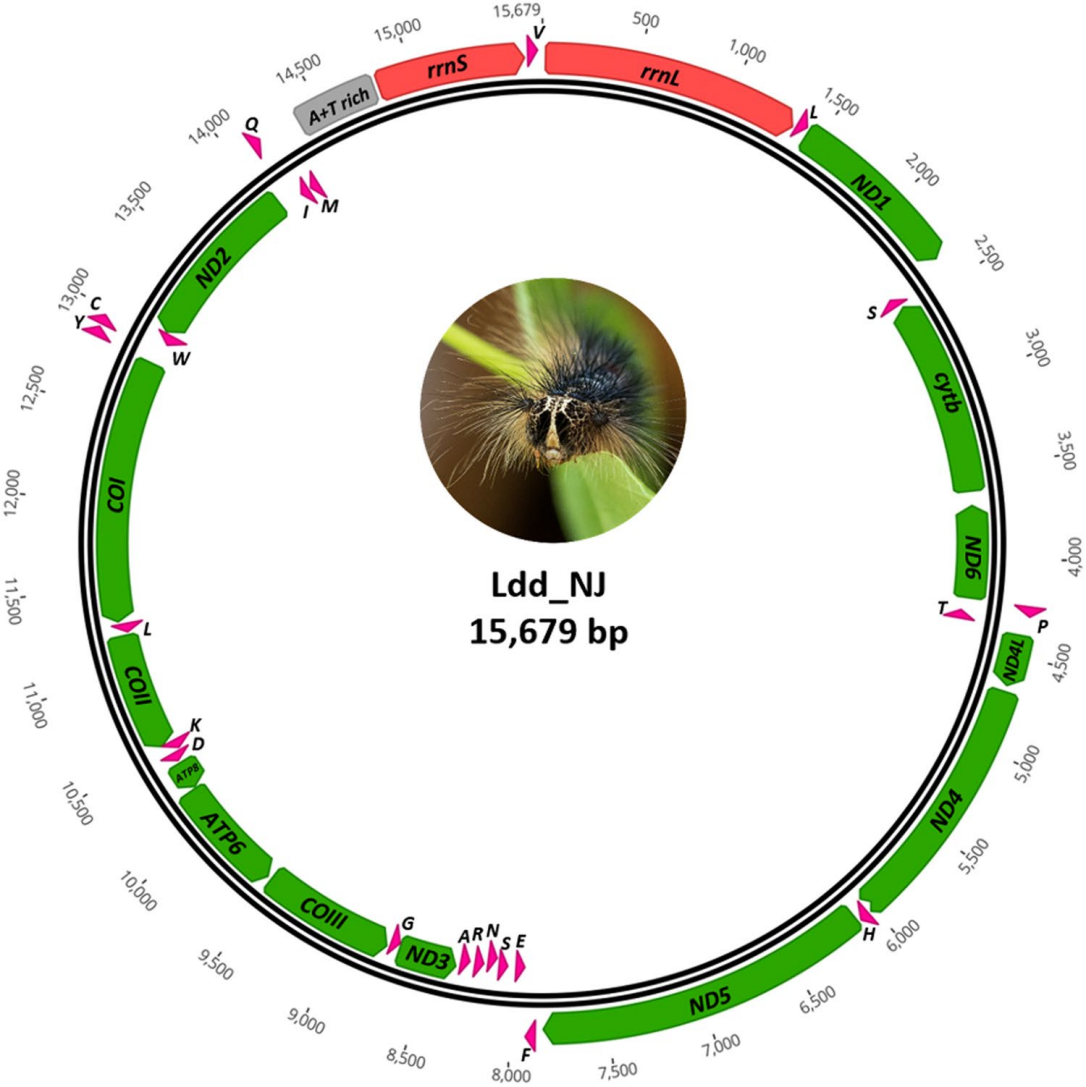
Circular map of mitochondrial genome of *L. dispar dispar* (Ldd_NJ). The tRNA genes are indicated using the single letter IUPAC-IUB abbreviation, corresponding to their amino acid. COI, II and III: cytochrome oxidase subunits; Cyt b: Cytochrome b; ND: NADH dehydrogenase; rrnL and rrnS rRNA correspond to ribosomal RNAs. Gypsy moth caterpillar photo credit: Catherine Béliveau^©^.

As expected, all 11 sequenced *Lymantria* mitochondrial genomes were of similar size (~15,600 bp; Table 2), falling within the size range of other lepidopteran mitogenomes sequenced earlier^15^. Most of the observed size variation was attributable to differences in the non-coding A + T-rich region, which is subject to a higher rate of mutation and/or rearrangement than other regions. Not surprisingly, all 11 mitogenomes yielded the same annotation, an example of which is provided in Fig. 2 for *L. dispar dispar*. As described for other insect mitogenomes^15,16^, the circular double-stranded DNAs contained a conserved set of 37 genes (Table 3), including 13 protein-coding genes (PCGs), 22 transfer RNA genes (tRNA), a large (16 S) and a small (12 S) ribosomal RNA subunit gene (*rrnL* and *rrnS* rRNA), and a large non-coding A + T-rich region, located between tRNA-Met and rrns rRNA (Fig. 2). The A + T content (Table 2) and the arrangement and orientation of genes (Fig. 2) were similar to those reported for other lepidopteran mitochondrial genomes^16,17^. All PCGs were observed to use an ATN start codon, except for the COI gene, which used CGA as start codon, as reported for other Lepidoptera^17^. The genomes presented here also showed incomplete stop codons (T- or TA-) for the ND4, COI and COII genes, as observed earlier for other lepidopteran mitogenomes^16,18^. There were no structural differences among the nine *L. dispar* mitogenomes analyzed here, but there was a codon deletion in the Lu_JH ATP8 gene.

**Table 3:** Organization of the *L. dispar dispar* mitochondrial genome.

Gene	Position (Min-Max)	Length (bp)	Start/Stop codon	Anticodon	Direction*
*rrnL*	12–1,366	1,355			F
*trnL1* (Leu)	1,367–1,435	69		TAG	F
*ND1*	1,436–2,374	939	ATA/TAA		F
*trnS2* (Ser)	2,403–2,471	69		TGA	R
*cytb*	2,474–3,634	1,161	ATG/TAA		R
*ND6*	3,701–4,255	537	ATA/TAA		R
*trnP* (Pro)	4,248–4,312	65		TGG	F
*trnT* (Thr)	4,313–4,377	65		TGT	R
*ND4L*	4,390–4,674	285	ATG/TAA		F
*ND4*	4,697–6,036	1340	ATG/TA-		F
*trnH* (his)	6,037–6,101	65		GTG	F
*ND5*	6,102–7,850	1,749	ATT/TAA		F
*trnF* (Phe)	7,876–7,941	66		GAA	F
*trnE* (Glu)	7,944–8,010	67		TTC	R
*trnS1* (Ser)	8,047–8,114	68		GCT	R
*trnN* (Asn)	8,114–8,178	65		GTT	R
*trnR* (Arg)	8,189–8,250	62		TCG	R
*trnA* (Ala)	8,273–8,338	66		TGC	R
*ND3*	8,343–8,699	354	ATT/TAA		R
*trnG* (Gly)	8,697–8,761	65		TCC	R
*COIII*	8,764–9,552	789	ATG/TAA		R
*ATP6*	9,564–10,241	678	ATG/TAA		R
*ATP8*	10,235–10,417	183	ATT/TAA		R
*trnD* (Asp)	10,418–10,486	69		GTC	R
*trnK* (Lys)	10,486–10,556	71		CTT	R
*COII*	10,537–11,238	682	ATA/T-		R
*trnL2* (Leu)	11,239–11,305	67		TAA	R
*COI*	11,301–12,854	1,531	CGA/T-		R
*trnY* (Tyr)	12,847–12,910	64		GTA	F
*trnC* (Cys)	12,917–12,982	66		GCA	F
*trnW* (Trp)	12,975–13,042	68		TCA	R
*ND2*	13,041–14,054	1,014	ATT/TAA		R
*trnQ* (Gln)	14,102–14,170	69		TTG	F
*trnI* (Ile)	14,175–14,241	67		GAT	R
*trnM* (Met)	14,242–14,308	67		CAT	R
A + T rich	14,369–14,816	448			—
*rrnS*	14,818–15,602	785			F
*trnV* (Val)	15,609–15,674	66		TAC	F

^*^F: forward (L-strand); R: reverse (H-strand).

### Comparative mitogenome analysis

#### Nucleotide sequences

**Figure 3:**
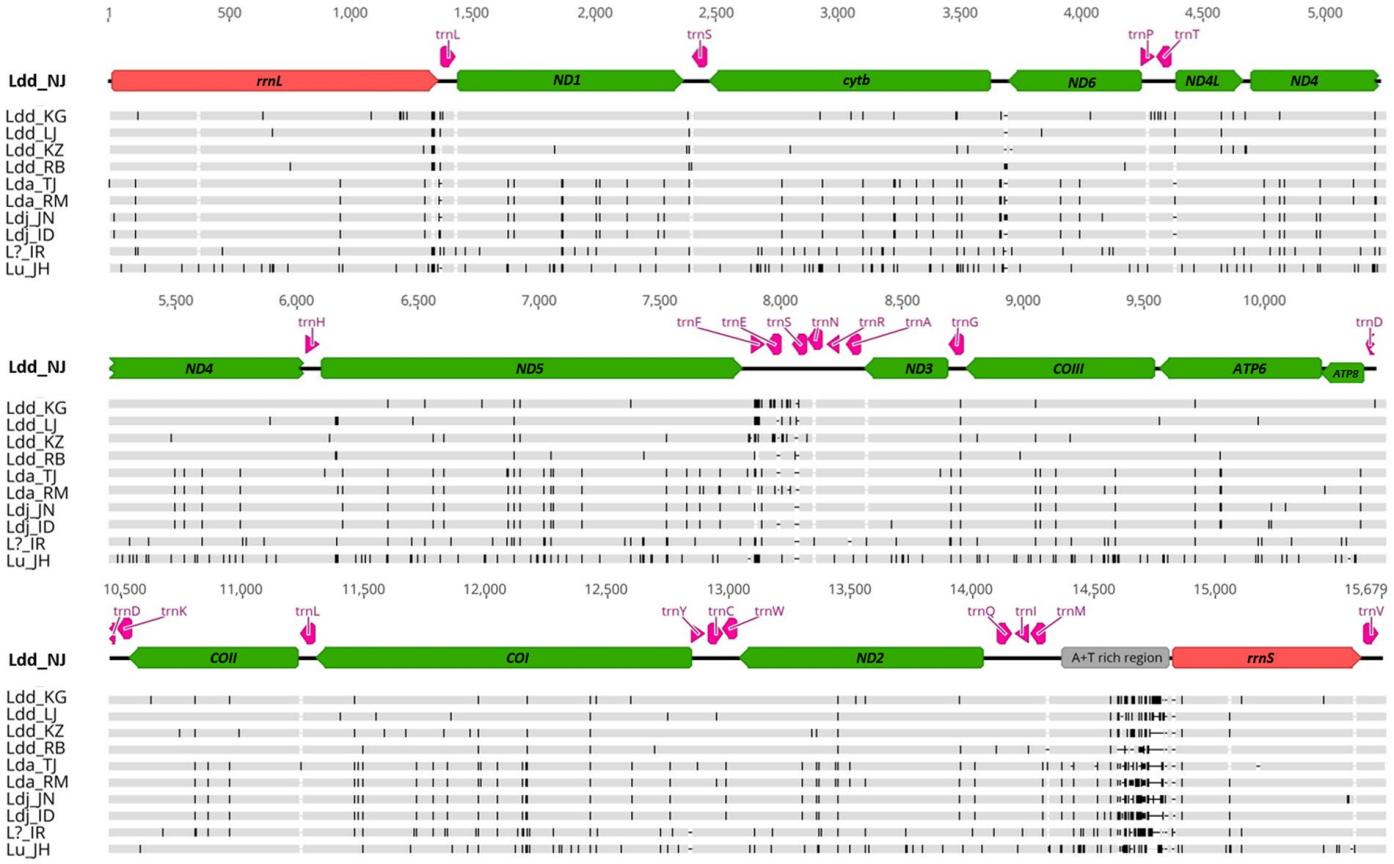
Graphical representation of a nucleotide alignment of the 11 mitochondrial genomes sequenced in the present study. Black vertical bars represent substitutions in the nucleotide sequence relative to the Ldd_NJ genome (top). Annotations: green boxes, PCGs; red boxes, rRNA subunit genes; grey box, A + T-rich region; pink arrows, tRNAs.

To assess variability and divergence among the 11 gypsy moth mitogenomes examined here, we conducted three different analyses. First, we aligned full genomic sequences to identify positions of single nucleotide polymorphisms (SNPs), using the genome of Ldd_NJ as reference (Fig. 3), while more quantitative assessments were obtained through computation of pair-wise percent identities (Table S2) and haplotype network analysis (Fig. S1). A simple visual examination of the graphical representation shown in Fig. 3 (see also Fig. S1) immediately brings to light the high degree of similarity among the four Asian *L. dispar* sequences, which comprise the *asiatica* and *japonica* subspecies, relative to the five *L. dispar dispar* geographic variants. In total 53 SNPs were common to all four Asian *L. dispar* mitogenomes, enabling easy discrimination between these two subspecies and *L. dispar dispar*. Despite the high sequence identity observed between *L. dispar asiatica* and *L. dispar japonica* (Table S2 and Fig. S1), SNPs that could tell them apart were identified in ND1, ND2, ND4, ATP6 and COI (Fig. 3; note that the latter was reported earlier^8^). The two *japonica* mitogenomes displayed fewer substitutions (4; Fig. S1) than their two *asiatica* counterparts (22; Fig. S1), perhaps reflecting differences in geographical distance between the two populations sampled for each subspecies.

The overall level of nucleotide identity among the five *L. dispar dispar* geographic variants was slightly lower than that observed among the four Asian samples (Table S2; Fig. S1), a conclusion that is congruent with the more scattered distribution of SNPs seen among the *L. dispar dispar* variants (Fig. 3). Some regions of the *L. dispar dispar* mitochondrial genomes sampled here, notably COI and ND5, displayed SNPs that may be diagnostic of the populations these insects were drawn from (Fig. 3).

The mitogenome of *L. umbrosa*, which was considered a subspecies of *L. dispar* until the taxonomic revisions of Pogue and Schaeffer^2^, displayed a large number of substitutions (243; Fig. S1) relative to the mitogenomes of *L. dispar* (Fig. 3; Table S2), providing additional support for the elevation of its status as a distinct species. In comparison, the gypsy moth sample from northern Iran displayed a number of substitutions (123; Fig. S1) intermediate between *L. umbrosa* and those identified as *L. dispar* (Fig. 3; Table S2). Although only one specimen was used to generate the full mitochondrial genome for that population, we sequenced a region spanning the COI and ND2 genes from four additional samples, including another one from northern Iran and three from the Russian Caucasus (Fig. S2). The almost perfect sequence identity observed among these five samples suggests that they belong to the same, genomically distinct population, found in the vicinity of the Caucasus.

### Amino acid sequences

**Figure 4:**
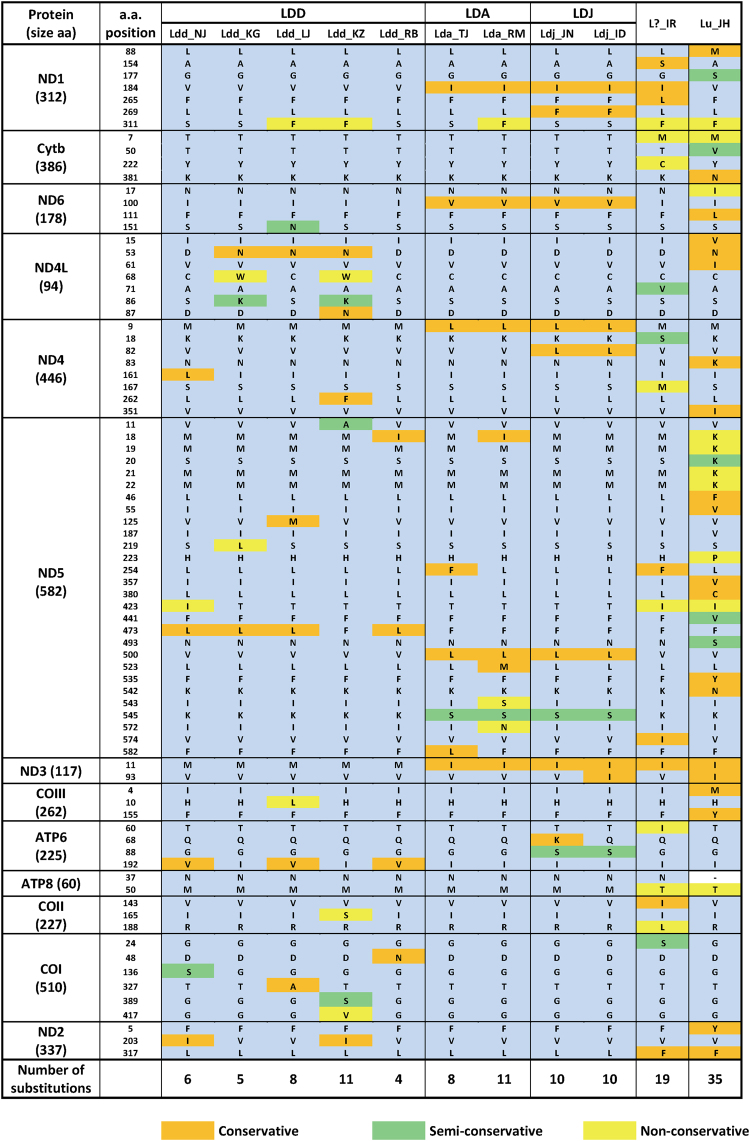
Comparative analysis of amino acid substitutions among the 13 PCGs of the 11 mitochondrial genomes sequenced in the present study. Unsubstituted amino acids at a given position are shaded in blue; substitutions are shaded in different colors according to their conservativeness (see legend). To identify substitutions and assess their level of conservativeness, amino acid sequences were aligned in MAFFT, using the ClustalW output format.

Following conceptual translation of the above DNA sequences, some of the SNPs we identified proved to be non-synonymous, resulting in amino acid substitutions. As those could have an impact on enzymatic activity and energy metabolism, possibly affecting flight capacity, we identified all amino acid substitutions among the 11 mitogenomes that we sequenced and determined whether they were conservative or not (Fig. 4). Not surprisingly, more amino acid substitutions were detected in the Lu_JH (35 substitutions) and L?_IR (19 substitutions) mitogenomes than in the remaining *L. dispar* genomes (average of 8 substitutions), using the most common cross-taxa residue as a point of reference (i.e., those shaded in blue in Fig. 4). Four substitutions were unique to the four Asian *L. dispar* mitogenomes (in ND4, ND5 and ND6), while two additional ones were shared by the Asian *L. dispar* mitogenomes and that of L?_IR (ND1) or those of both L?_IR and Lu_JH (ND3); all but one (K→S in ND5) of these substitutions were deemed conservative (Fig. 4). Three substitutions were unique to the *L. dispar japonica* mitogenome (in ND1, ND4 and ATP6), including one considered semi-conservative (G→S in ATP6). Among the *L. dispar dispar* mitogenomes, there were several scattered substitutions including some non-conservative ones, but no clear pattern could be detected. Importantly, no amino acid substitutions were common to the four Asian *L. dispar* populations and those with an *L. dispar dispar* mitochondrial signature but whose females display flight capability (Ldd_LJ and Ldd_RB^5^), considerably decreasing the likelihood of a hypothetical substitution that would confer an energetic advantage to populations with flight-capable females.

### Phylogenetic analyses

**Figure 5:**
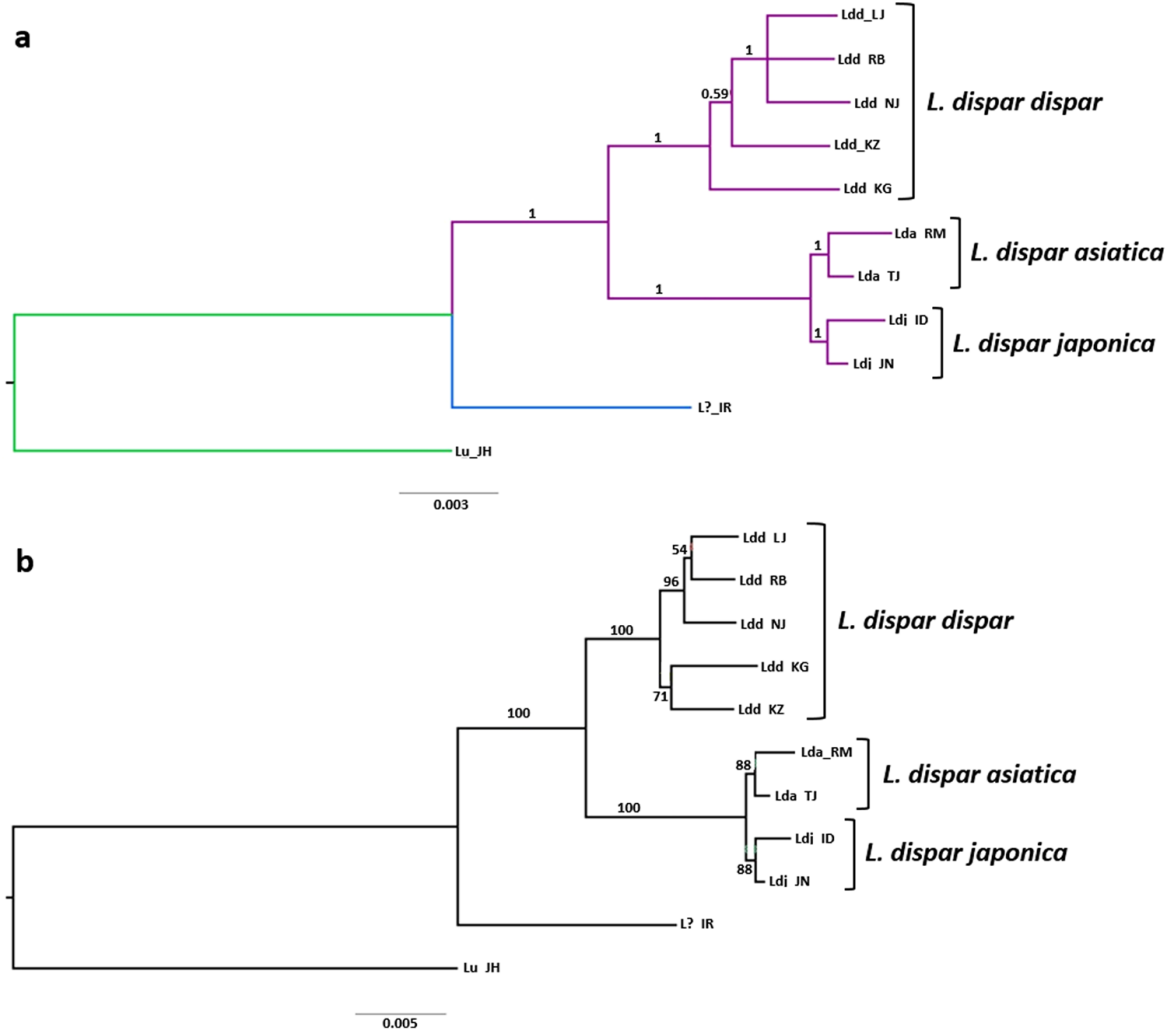
Phylogenetic relationships among the nine *L. dispar* populations sampled here, plus a population from Iran (L?_IR) and *L. umbrosa*, based on all 37 mitochondrial genes, plus the A + T-rich region. (**a**) Bayesian inference (BI), MCMC consensus tree (10,000,000) with posterior probabilities shown on each branch. (**b**) Maximum Likelihood (non-partitioned data) with 1000 bootstrap replicates. *L. umbrosa* was used as outgroup.

All analyses conducted here yielded the same topology for a given tree building approach (Maximum Likelihood [ML] and Bayesian inference [BI]), irrespective of the dataset (PCGs alone, PCGs + tRNA + rRNAs or PCGs + tRNA + rRNAs + A/T-rich region), with and without partitioning schemes, although there were differences in bootstrap (in ML) and posterior probability (in BI) support values among terminal taxa. None of the PCGs were found to be under positive selection and all but two (ND3 and ND4L) were considered to be under purifying selection (Table S3), as is expected for mitochondrial PCGs^19^. Figure 5 shows the BI and ML trees obtained using the full data set, with (BI) and without (ML) partitioning scheme.

In both BI and ML analyses, the representative from northern Iran formed a sister group to all *L. dispar* lineages, assuming a position intermediate between *L. dispar* and *L. umbrosa*. Indeed, the monophyly of *L. dispar* was not supported when the specimen from Iran was included in the analysis. Relationships among the three recognized subspecies were clear, although the two Asian subspecies formed a monophyletic group, separated by very short branches, but with strong branch support in both analyses (Fig. 5). The two analyses placed the European specimens within a strongly supported clade (bootstrap = 100; posterior probability = 1), but with unstable and poorly resolved internal relationships, where the Greek and Kazakhstan specimens formed a distinct, moderately supported clade (bootstrap = 71) in the ML tree, and the Greek specimen was basal to the rest of the group in the BI tree (Fig. 5).

## Discussion

This study represents the first detailed analysis of multiple gypsy moth mitogenomes, featuring a taxon sample that comprises all three *L. dispar* subspecies and geographical variants thereof, plus the closely related *L. umbrosa* and a previously uncharacterized gypsy moth population displaying a mitochondrial haplotype similar to, but evidently distinct from, that of *L. dispar*. Previous molecular comparisons of gypsy moth variants relied primarily on the barcode region of the COI gene, which is here shown to be rich in nucleotide substitutions, some of which are useful for subspecies delineation. However, we found other mitochondrial genes to contain many additional informative SNPs with potential for segregating *L. dispar dispar* from the two Asian subspecies and for distinguishing *L. dispar asiatica* from *L. dispar japonica* (Fig. 3), which cannot be told apart using the barcode region alone^10^. Interestingly, ND2 and ATP6, two genes that we found to contain informative SNPs for this purpose, were among the five mitochondrial genes sampled by Wu *et al*.^12^ to generate a phylogeny of *L. dispar* variants in which the two Asian subspecies formed distinct clades.

Although the present analysis did not include multiple individuals from each of the populations sampled, thereby making it difficult to assess haplotype homogeneity within any given population, the number of SNPs observed among the five *L. dispar dispar* mitogenomes (Fig. 3) was large enough to justify the prediction that these genomes are likely to contain markers that will provide useful clues for identifying the geographic origins of unknown samples. The fact that more variability was observed among the five *L. dispar dispar* mitogenomes than among those of the four Asian samples, which comprise two subspecies, is not entirely surprising given the much greater geographic distance separating the *L. dispar dispar* populations than that separating the other four (Fig. 1). Within the *L. dispar asiatica* subspecies, COI-based evidence has recently been presented in support of population-specific mitogenome differences among samples obtained from different locations in China^11^, strongly suggesting that a broad geographic sampling of *L. dispar asiatica* would reveal inter-population differences similar to those observed here for *L. dispar dispar* mitogenomes. Thus, future population genomics studies aimed at developing markers for the purpose of identifying geographic origins should consider both nuclear and mitochondrial genomes.

Although mitochondrial markers have achieved great success for taxon delineation, their use for gypsy moth subspecies identification has proven somewhat tenuous when genotyping populations found primarily in central Asia, which display an *L. dispar dispar* COI haplotype^8^, but for which nuclear markers^8,12,13^ and phenotypes, including female flight capability^5^, suggest they have more in common with the *asiatica* than with the *dispar* subspecies. In fact, insects from some of these populations appear to have a *dispar/asiatica* mosaic nuclear genome^13^ (see also populations from Kazakhstan and Kyrgyzstan in^12^), suggestive of hybridization between the two subspecies, presumably after a period of reproductive isolation. Although we have not yet produced nuclear gene-based phylogenies equivalent to the ones presented here using mitogenomes (Fig. 5), the likelihood of discordance between such phylogenies for central Asian populations is high. This type of discordance is common^20^ and, in the case at hand, raises the question as to why central Asian hybrids would have a *dispar* instead of an *asiatica* mitogenome haplotype (note: it is not clear whether all populations in central Asia have a *dispar*-like mitogenome, but all those examined by our group do; see^8^).

Given that mitochondrial genomes are typically of maternal inheritance, the biogeographic discordance between nuclear and mitochondrial genomes observed in moths from central Asia could have resulted from (i) male-biased dispersal, resulting in a higher likelihood of crosses between *L. dispar asiatica* males and *L. dispar dispar* females than for the reciprocal cross^21^, and/or (ii) adaptive introgression of mitogenomes that strongly favors one mitogenome over the other^20^. If, as shown for other lepidopteran species^21^, gypsy moth males are the more mobile sex, irrespective of whether females are capable of flight or not, the situation observed here for central Asian populations suggests that *L. dispar asiatica* males could have colonized the range of *L. dispar dispar*, where asymmetric introgression took place from the local *dispar* population into the colonizing *asiatica* population, leading to the observed preponderance of the *dispar* mitogenome haplotype in such hybrids (see^21^). In addition, since mitochondrial OXPHOS proteins are subunits of larger proteins featuring both mitochondrial and nuclear components (N-mt genes), amino acid substitutions within these subunits can negatively affect protein assembly and lead to mitonuclear incompatibilities^22^. Alternatively, some mitochondria can accumulate a significant number of mildly deleterious mutations that are rescuable by replacement with a more fit haplotype^20^. Whether the latter scenario played a role in the mitochondrial haplotype pattern observed here is unknown. Although we found a significant number of amino acid substitutions among *L. dispar* mitogenomes, the available data do not permit predictions regarding levels of mitonuclear compatibility since the sequences of the N-mt genes are not currently available. Cointrogression of N-mt genes from *L. dispar dispar* into the central Asian genomes (featuring an otherwise *asiatica* genetic background) could also have favored the maintenance of the *dispar* mitogenomes in these populations^20^, a hypothesis that will be worth examining once the gypsy moth nuclear genome has been sequenced and annotated.

Interestingly, crosses between females of *L. umbrosa* (previously known as *L. dispar praeteria*) and males of *L. dispar japonica* yielded all-male broods, a result the authors attributed to some genetic incompatibility between the two taxa^23^. In view of our observation that the *L. umbrosa* mitogenome features 32 amino acid substitutions relative to that of *L. dispar japonica*, including 9 substitutions that are considered non-conservative (Fig. 4), the question arises as to whether the reported unviability of hybrid females^23^ could be explained, at least in part, by an incompatibility between a recessive paternal Z chromosome and maternal cytoplasm (e.g., mtDNA)^24^. Indeed, this type of female hybrid unviability fits Haldane’s rule, which predicts unviability of hybrids of the heterogametic sex (female in the case of Lepidoptera)^24^.

Irrespective of the method used, the phylogenetic analyses presented here (Fig. 5) generated trees for which the overall *L. dispar* branching pattern was similar to that obtained by Wu *et al*.^12^ using five mitochondrial genes (ND2, ND6, ATP6, ATP8 and Cytb), where the two Asian subspecies formed distinct, but more derived clades. In the latter study, the tree showed *L. dispar dispar* samples from the United States forming varying associations with those from France, Germany and Italy. However, that analysis did not include *L. dispar dispar* mitochondrial haplotypes from central Asia. Here, the North American haplotype clustered with those from Lithuania and Siberia, as opposed to that from Greece (i.e., closer to the presumed origin [France] of North American moths), which appeared basal to the *L. dispar dispar* group in the BI analysis and forming a basal cluster with the sample from Kazakhstan in the ML analysis (Fig. 5). A wider and finer sampling scheme across the entire range of *L. dispar dispar* mitochondrial haplotypes would provide a better assessment of connectivity among haplotypes.

As expected from the number of SNPs found in its mitogenome (i.e., compared with *L. dispar* and the population from northern Iran; Fig. 3), *L. umbrosa* occupied a basal position in the phylogenetic trees presented here, while the previously uncharacterized sample from Iran occupied an intermediate position between *L. umbrosa* and the *L. dispar* clade (Fig. 5). The latter observation raises important questions regarding the taxonomic status of this “Caucasian” gypsy moth population and its role in the evolutionary history of the *L. dispar* lineage. We currently have no information on biological attributes that may help distinguish this population from other gypsy moth populations, including morphometrics, female flight capability and host preference. A scan of the gypsy moth literature published in the USSR prior to 1991 (compiled in^25^) identified many publications devoted to studies of gypsy moth populations in the Caucasus (Russian Caucasus, Georgia, Armenia and Azerbaijan); however, because *L. dispar* and the “Caucasian” population may well be sympatric (and treated without distinction by earlier workers), no conclusion can be readily drawn from this literature. Thus, before establishing the taxonomic status of this population, i.e. whether it should be treated as a new subspecies of *L. dispar* or a distinct species altogether, additional information will need to be gathered on its unique biological traits, if any. In addition, a nuclear marker-based genomic comparison with gypsy moth samples identified as *L. dispar* will need to be conducted.

As to why the “Caucasian” population has such a distinct mitochondrial haplotype relative to *L. dispar*, important physical barriers such as the Greater and Lesser Caucasus Mountains, which form a deep valley in Azerbaijan, could have kept it in complete reproductive isolation for a long period, after which it may have expanded its range through paths along the Caspian and Black Seas. A similar type of reproductive isolation has been proposed as the mechanism that drove differentiation between the “Hokkaido gypsy moth” (*L. umbrosa*) and *L. dispar asiatica*. Although they are now reunited (creating zones of sympatry between the two species), the eastern portion of the Japanese island of Hokkaido was once separated by water from its western segment, thereby creating a reproductive barrier that appears to have led to the speciation of *L. umbrosa* ^2^.

Finally, although we cannot as yet establish whether the “Caucasian” population examined here is of significant biosecurity concern to North America, shipping vessels traveling from ports of the Black Sea could inadvertently carry these insects and, in doing so, favor their accidental introduction into Canada and the United States, therefore justifying the use of molecular tools to properly identify them. At the time we developed our TaqMan qPCR assay targeting Asian gypsy moths and related species posing a threat to North America^8^, we had no knowledge of the “Caucasian” population; as a consequence, it was not considered in the design of our assay. Here, when the five “Caucasian” samples were submitted to the TaqMan assay, they were all incorrectly identified as *L. dispar dispar*. Thus, we designed a new version of the “Simplex 3” sub-assay (corresponding to the “Is it Ldd?” node of Fig. 1 in^8^) to enable discrimination between *L. dispar dispar* and the “Caucasian” strain. To this end, we used SNPs within ND1 identified in the present work (manuscript in preparation).

## Materials and Methods

### Sampling strategy

Based on currently established criteria for the taxonomic placement of *L. dispar* and its subspecies^2,26^, we sampled one adult male from each of 10 populations distributed over the gypsy moth’s range, including five geographic variants of *L. dispar dispar*, two exemplars each of *L. dispar asiatica* and *L. dispar japonica*, and one specimen from northern Iran deemed to be of uncertain taxonomic affiliation (originally identified as *L. dispar*), together with the sister species *L. umbrosa*, used here as outgroup taxon (Table 1; Fig. 1). Sources of samples are listed in Table 1. To confirm identifications made based on morphological features, we used two molecular taxonomic tools: a multi-species TaqMan assay^8^ and DNA barcoding^9^. For the latter method, specimens were cross-checked with their DNA barcodes (COI 5′), as found in the BOLD (Barcode of Life Data System) database^27^, which contains a reference library for global gypsy moth populations, including the geographic variants used in this study. Two *L. dispar* specimens from central Asia (i.e., specimens 4 [Kazakhstan] and 5 [Russia; Siberia] as shown in Fig. 1) with uncertain subspecies affiliation are here treated as *L. dispar dispar* based on their mitochondrial haplotypes (Table 1).

### DNA extraction

Total genomic DNA (nuclear and mitochondrial) was isolated from specimens that were either dry, preserved in 96% ethanol or frozen, using either the Qiagen Blood & Cell Culture DNA midi kit or the Qiagen DNeasy Blood & Tissue mini kit (Qiagen, Hilden, Germany), following the manufacturer’s instructions. For frozen material, the specimen’s wings and abdomen were first removed and the remaining body parts were ground in liquid nitrogen prior to DNA extraction as described^8^. For archival specimens, one or more moth legs were collected, ground in liquid nitrogen and submitted to DNA extraction with the following minor modifications: 56 °C incubation was conducted overnight and final DNA elution was done in 100–150 μL of sterile water. Eluted DNA was then quantified using a NanoDrop ND-1000 spectrophotometer (Thermo Fisher Scientific Inc.) and used either directly or diluted for mitochondrial genome PCR amplification.

### Primer design, PCR amplification and sequencing

An initial assembly of the Ldd_NJ mitogenome was gleaned from a Newbler assembly generated during the sequencing of the *L. dispar dispar* nuclear genome, using Roche 454 sequencing technology. Similarly, initial assemblies of the Lda_TJ and Ldj_ID mitogenomes were obtained from nuclear genome assemblies generated using the Celera assembler on PacBio sequencing reads. Following error correction (see below), these three mitogenomes were used as a reference for the assembly of the mitochondrial genomes of the other specimens analyzed in this study.

To perform error correction on the three above mitogenomes and to obtain the sequences of the eight remaining genomes (Table 1), two specific primer pairs (P1533/P1365 and P1364/P1532, see Table S1) were designed (based on a multiple alignment of conserved regions of COI, Cytb and ND6) to amplify two long fragments (fragment 1: COI→Cytb and fragment 2: ND6→COI, 8,949 and 7,048 bp in size, respectively) overlapping in the ND6 region. Sequencing of different portions of these long fragments was achieved by either submitting these fragments directly to Sanger sequencing or by conducting prior amplification of shorter, overlapping portions using specific primer pairs (Table S1). All PCR amplifications were performed directly on total DNA extracts (0.5–5 ng) in a 25 µL final volume, using 0.5 U of Platinum SuperFi DNA polymerase (Invitrogen), 1x SuperFi buffer (Invitrogen), 0.33 mM of a dATP/dTTP mix, 0.10 mM of a dCTP/dGTP mix, 10 pmol of each primer and 9% DMSO. PCR conditions were: initial denaturation step at 95 °C for 2 min, followed by 40 cycles of denaturing (95 °C, 5 sec), coupled annealing and extension (60 °C, 10 min), and a final extension step at 60 °C for 10 min. Smaller fragments were also amplified to help resolve regions that were difficult to sequence (Table S1). One µL of PCR product was then analyzed by gel electrophoresis, and the remaining reaction volume used directly for Sanger sequencing. Primers used for the latter purpose were either those designed for PCR amplification or new ones especially designed for sequencing (see Table S1 for the complete list of primers used). Sequencing was performed by the Genome Sequencing and Genotyping platform of the CHUL, in Quebec City (Quebec, Canada).

### Sequence assembly and genome annotation

Assembly and annotation were carried out using Geneious® software, version 9.1.2 (http://www.geneious.com)^28^, in combination with the NCBI Blast function to help predict PCGs. Transfer RNA genes were identified using tRNAscan-SE 1.21^21,22,29,30^, with the search mode and the cove cutoff score set as default, Mitochondria/Chloroplast as the search source and invertebrate mitochondrial genome as the genetic code for tRNA isotype prediction.

### Assessment of the extent to which mitochondrial PCGs are under positive selection

To determine whether the mitochondrial PCGs considered in the present study were under positive selection, we used the codon based z-test, which compares relative abundances of synonymous and nonsynonymous mutations, as implemented in Mega 7.0^31^.

### Phylogenetic analyses

Matrices of mitogenomic data were analyzed using two model-based phylogenetic approaches, namely Maximum Likelihood (ML) and Bayesian Inference (BI). To explore phylogenetic signal in the data, the effects of varying gene combinations were first compared against analyses that included the full dataset, either concatenated or partitioned according to genes. On the basis of these preliminary explorations, we decided to include all genes and third codon positions in both ML and BI analyses; similarly, we decided to use non-partitioned data for ML analysis and partitioned data (1 partition for each gene; 38 partitions) for BI analysis. We found that inclusion of all genes/regions, including 13 protein-coding genes (PCGs), 22 transfer RNA genes (tRNA), large (16S) and small (12S) ribosomal RNA subunit genes (*rrnL* and *rrnS* rRNA), and the large non-coding A + T-rich region, together with a partitioning scheme for BI, improved bootstrap (in ML) and posterior probability (in BI) support values among terminal taxa. BI and ML analyses were performed using the software MrBayes v3.1^32^ and the recently developed IQ-TREE^33^, respectively. IQ-TREE searches were carried out using the default settings on the dedicated web-server IQ-TREE, available at http://iqtree.cibiv.univie.ac.at/ ^34^; the most appropriate models of sequence evolution were chosen using the Auto function on the IQ-TREE web server, following the authors’ recommendations. ML bootstrap analysis^35^ and clade robustness were then assessed using ultrafast bootstrap replicates with IQ-TREE. The Bayesian analyses were run independently twice for 10 million generations, with every 1000th tree sampled. The data matrix was split into 38 partitions: AT_rich, ATP6, ATP8, COI, COII, COIII, cyt-b, ND1, ND2, ND3, ND4, ND4L, ND5, ND6, rrnL, rrnS, trnA, trnC, trnD, trnE, trnF, trnG, trnH, trnI, trnK, trnL-1, trnL-2, trnM, trnN, trnP, trnQ, trnR, trnS-1, trnS-2, trnT, trnV, trnW, and trnY. We modelled the evolution of sequences according to the GTR + Γ model independently for the 38 partitions using the “unlink” command in MrBayes. Clade support was estimated by posterior probabilities in MrBayes. Convergence was determined when the average standard deviation of split frequencies (ASDSF = 0.013558) went below 0.05, the PSRF (Potential Scale Reduction Factor) approached 1, and both runs had properly converged to a stationary distribution after the burn-in stage (which was 1,000 sampled generations; burn-in = 7000).

### Haplotype network analysis

Median joining networks were computed to examine intra- and interspecific divergence among the 11 mitogenomes examined here using default settings, as implemented in the software popart (Population Analysis with Reticulate Trees)^36^.

### Data availability

All sequences reported in this paper have been deposited in GenBank. Accession numbers are provided in Table 2.

## Electronic supplementary material

Supplementary material
