# Supplementary material for: Comparative analysis of mitochondrial genomes of geographic variants of the gypsy moth, Lymantria dispar, reveals a previously undescribed genotypic entity
Source: Sci Rep. 2017 Oct 27;7:14245. doi: 10.1038/s41598-017-14530-6 (PMC5660218; doi:10.1038/s41598-017-14530-6)
Supplement: Supplementary file 1 — Supplementary material [file 41598_2017_14530_MOESM1_ESM.pdf]

Supplementary material for the manuscript:

Comparative analysis of mitochondrial genomes of geographic variants of the gypsy moth, *Lymantria dispar*, reveals a previously undescribed genotypic entity

by

Abdelmadjid Djoumad, Audrey Nisole, Reza Zahiri, Luca Freschi, Sandrine Picq,

Dawn E. Gundersen-Rindal, Michael E. Sparks, Ken Dewar, Don Stewart, Halim Maaroufi,

Roger C. Levesque, Richard C. Hamelin, & Michel Cusson

Scientific Reports

Table S1. List of primers used to amplify and sequence the mitochondrial genomes.

Name	Sequence	Position on Ldd_NJ	Length	Direction	Fragment
P1577_16S-F	GGTATTTTAAAGGAATTCGGC	633	21	F	2
Alb_16SrRNA	TGATTATGCTACCTTTGTACAGTCA	753	25	R	2
P1525inv	CTGCCAAAATAGTAAATCCAT	1,359	21	R	2
P1525_ND1	ATGGATTTACTATTTTGGCAG	1,359	21	F	2
P1519inv	CTCCTGTATTAGCTTTTGCT	1,677	20	F	2
P1519_ND1	AGCAAAAGCTAATACAGGAG	1,677	20	R	2
P1554_Post_ND1	TACTCTACCTCGTTATCGTTAT	2,260	22	F	2
P1524_Cytb	TGATCCAGTTTGATGAAGAA	2,999	20	F	2
P1533inv	CATATTGGACGAGGAATTTATTATGAATC	3,297	29	R	2
P1533_ND6*	GATTCATAATAAATTCCTCGTCCAATATG	3,297	29	F	2,1
P1553_Post_Cytb	GGTTTGATTTTCGTATAAGAATGAAT	3,589	26	F	2,1
P1552_Post_Cytb	ATTCATTCTTATACGAAAATCAAACC	3,589	26	R	2,1
P1556_Alb_Cytb	ATATTGGTAATTTTGACTACTGC	3,735	23	F	2,1
P1532_ND6*	GGTCTTTTAGTTTTATTTATTTATGTTTCAAGAATTGC	4,023	38	R	2,1
P1532inv	GCAATTCTTGAAACATAAATAAATAAAACTAAAAGACC	4,023	38	F	1
P1522inv	AATCCTATAGAAAGGGGGTT	4,158	20	F	1
P1522_ND6	AACCCCCTTTCTATAGGATT	4,158	20	R	1
P1545_ND4	AATTAAAGTAGGAATTAAAC	5,052	20	R	1
P1544_ND4	CATCTAATATGGCTGCYCCTC	5,751	21	F	1
P1528inv	TACCTGAATAACTTTCTCCATG	5,906	22	R	1
P1528_ND4	CATGGAGAAAGTTATTCAGGTA	5,906	22	F	1
P1527_ND5	TTCTTCAGTGGTTATTTAT	6,314	19	F	1
P1521_ND5	AACATCCCGGCATTATATGA	6,513	20	R	1
P1530_ND5	CACCTACTCCAGTTTCTGCT	6,742	20	F	1
P1535_ND5	CACCTGCTGTAACTAAAGTAGAAG	6,772	24	R	1
P1536_ND5	CAAATATAGCTTTATGTGGRATTCC	7,150	25	F	1
P1529_ND5	CCTCACTTCATCCTATATCAAT	7,680	22	R	1
P1531inv	TCGAGCTGAAGATTTTGGATCA	8,547	22	F	1
P1531_ND3	TGATCCAAAATCTTCAGCTCGA	8,547	22	R	1
Ldja_COIII-R	TGAAGCCGCAGCATGATATTG	8,825	21	F	1
Ldja_COIII-F	CAATATCATGCTGCGGCTTCA	8,825	21	R	1
KazUmbl_COIII-R	ATTTGAAATGGRTTAAAGGGGATA	9,164	24	R	1
KazUmbl_COIII-F	TATCCCCTTTAAYCCATTTCAAAT	9,164	24	F	1
P1523inv	CAGTTGCGGTTATCCAATCA	9,612	20	R	1
P1523_ATP6	TGATTGGATAACCGCAACTG	9,612	20	F	1
P1557_Alb_ATP6	GAACTCCTCCTGCTTTAATA	9,822	20	R	1
R-COII	TGACTGAAAGTAAGTAATGGTCTC	10,526	27	F	1
P1559_Xyl_COII-F	GAGACCATTACTTGCTTTCAGTCATCT	10,526	24	R	1
2Alb_COII-R	AGACCATTACTTACTTTCAGCCA	10,527	23	F	1

P1518_COII	GCGGAACTAACCACAGATTT	10,621	20	R	1
Alb_COII	TGATGCTAACCCAGGACGAT	10,704	20	R	1
P1547_Post_COII	TGATGCCAACCCAGGACGAT	10,704	20	R	1
P1558_Alb_COI	TAGGTGTTAAAATTGATGCTAAC	10,714	23	R	1
P1562_Alb_COII	CCTCTCTCCGACTTCTATAT	10,984	20	R	1
P1549_Post_COII	TGTTCTATTAGAGGTGATGCTC	11,183	22	F	1
P1550_Post_COII	AGGTGATGCTCTATTTTGGAGAT	11,194	23	F	1
P1365*	CCTGTTTTAGCAGGTGCTATTAC	12,226	23	R	1
P1364 *	GATCATTTCCGATTAATGATCCAGG	12,692	25	F	2
Alb_COI	TACCGCTTAAACTCAGCCAT	12,853	20	R	2
Xyl_COI	TACAATTTATCGCTTAAACTCAGCCA	12,854	26	R	2
P1526_ND2	ATTGCAAATTTTAAGGAGTATTT	12,943	23	F	2
P1379_ND2	AATCCAATAAATGGGGGTAATCCTCCT	13,291	27	F	2
P1378inv	ATCCTTCAACAATATTGGGGAATCAA	13,694	26	F	2
P1378_ND2	TTGATTCCCCAATATTGTTGAAGGAT	13,694	26	R	2
ND2-F	TGGATGTTGAATTGGGTTAGA	13,945	21	F	2
P1520_ND2	TGGATGTTGAATTGGRTTAGA	13,945	21	R	2
P1536inv_ND2	CAAATATAGCTTTATGTGGRATTCC	13,945	21	R	2
Xyl_16SrRNA	AGAATAATCCTTTAATCAGGCACT	14,213	24	F	2
Alb_ND2tRNA	GATTTCCTTTATAGTTGGGGTATG	14,254	24	F	2
P1576_16S-R	ATATGAAAGCGACGGGCAATATGTAC	15,505	26	R	2

*Primers used to amplify the two main (initial) mitochondrial fragments

Table S2. Pair-wise percent identity among the 11 mitochondrial genomes sequenced. Values provided for the entire genome (bottom half, black letters) and for the protein coding genes (PCGs) only (upper half, red letters).

	PCGs										
	L. d. dispar					*L. d. asiatica*		*L. d. japonica*			
Complete mitogenome	Ldd_NJ	Ldd_KG	Ldd_LJ	Ldd_KZ	Ldd_RB	Lda_TJ	Lda_RM	Ldj_JN	Ldj_ID	L?_IR	Lu_JH
Ldd_NJ	-	99.7	99.8	99.7	99.8	99.2	99.2	99.3	99.3	98.9	97.9
Ldd_KG	99.0	-	99.6	99.6	99.6	99.2	99.2	99.3	99.3	98.9	97.8
Ldd_LJ	99.4	99.3	-	99.6	99.8	99.1	99.1	99.2	99.2	98.8	97.9
Ldd_KZ	99.4	99.3	99.4	-	99.6	99.2	99.2	99.2	99.2	98.8	97.9
Ldd_RB	99.0	98.6	99.0	99.1	-	99.2	99.2	99.3	99.3	98.9	97.9
Lda_TJ	98.6	98.4	98.4	98.7	98.9	-	99.8	99.9	99.9	98.8	97.6
Lda_RM	98.6	98.3	98.4	98.7	98.9	99.6	-	99.8	99.8	98.8	97.6
Ldj_JN	98.7	98.4	98.5	98.8	98.9	99.6	99.5	-	99.8	98.8	97.6
Ldj_ID	98.8	98.5	98.6	98.9	98.9	99.6	99.5	99.6	-	98.8	97.6
L?_IR	98.4	98.3	98.5	98.6	98.4	98.4	98.3	98.5	98.5	-	97.9
Lu_JH	97.7	97.7	97.9	97.8	97.5	97.3	97.3	97.4	97.4	97.7	-

Table S3. Assessment of whether each of the 13 mitochondrial protein-coding genes are under purifying or positive selection. At a significance level of 0.05, all but two genes were observed to be under purifying selection (blue highlighting), and none under positive selection.

Gene	Purifying (p-value)	Positive (p-value)
COI	<0.001	1
COII	0.017	1
COII	<0.001	1
ATP6	0.001	1
ATP8	0.016	1
Cytb	<0.001	1
ND1	<0.001	1
ND2	<0.001	1
ND3	0.065	1
ND4	<0.001	1
ND4L	0.302	1
ND5	<0.001	1
ND6	0.006	1

Figure S1

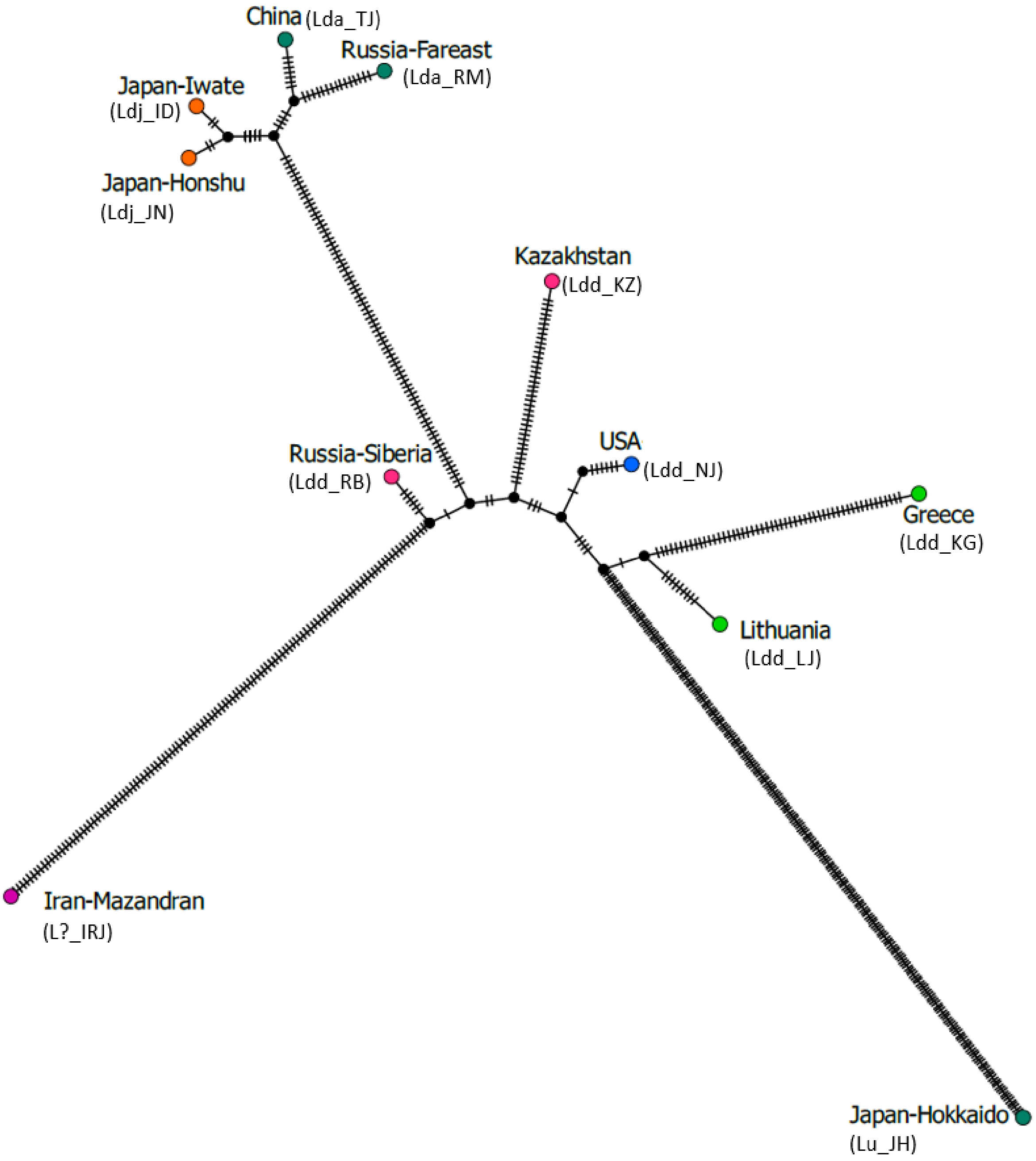

Figure S1. Haplotype network construction for the 11 mitochondrial genomes examined in the present study, carried out using POPART[35]. Each hatch mark represents one substitution.

Figure S2

A

```
                                                                           c
Lep0813_Iran      gcatgagctgtaacaatagtattataaatttgatcatttccgattaatgatccaggattt
Lep0816_Iran      gcatgagctgtaacaatagtattataaatttgatcatttccgattaatgatccaggattt
Lep0941_Russia    gcatgagctgtaacaatagtattataaatttgatcatttccgattaatgatccaggattt
Lep0944_Russia    gcatgagctgtaacaatagtattataaatttgatcatttccgattaatgatccaggattt
Lep0963_Russia    gcatgagctgtaacaatagtattataaatttgatcatttccgattaatgatccaggattt
                  ************************************************************

Lep0813_Iran      cctaattcagctcgaattagtaaacttagagatgttcccactattcctgatcaaatacca
Lep0816_Iran      cctaattcagctcgaattagtaaacttagagatgttcccactattcttgatcaaatacca
Lep0941_Russia    cctaattcagctcgaattagtaaacttagagatgttcccactattcctgatcaaatacca
Lep0944_Russia    cctaattcagctcgaattagtaaacttagagatgttcccactattcctgatcaaatacca
Lep0963_Russia    cctaattcagctcgaattagtaaacttagagatgttcccactattcctgatcaaatacca
                  **********************************************.*************

Lep0813_Iran      aaaataaaatataaggttccaatatccttatgatttgttgaatataatcattttcgctta
Lep0816_Iran      aaaataaaatataaggttccaatatccttatgatttgttgaatataatcattttcgctta
Lep0941_Russia    aaaataaaatataaggttccaatatccttatgatttgttgaatataatcattttcgctta
Lep0944_Russia    aaaataaaatataaggttccaatatccttatgatttgttgaatataatcattttcgctta
Lep0963_Russia    aaaataaaatataaggttccaatatccttatgatttgttgaatataatcattttcgctta
                  ************************************************************

Lep0813_Iran      ttttttaataaaatggctgagtttaagcggtaaattgtaaatttattaacgagaaatttc
Lep0816_Iran      ttttttaataaaatggctgagtttaagcggtaaattgtaaatttattaacgagaaatttc
Lep0941_Russia    ttttttaataaaatggctgagtttaagcggtaaattgtaaatttattaacgagaaatttc
Lep0944_Russia    ttttttaataaaatggctgagtttaagcggtaaattgtaaatttattaacgagaaatttc
Lep0963_Russia    ttttttaataaaatggctgagtttaagcggtaaattgtaaatttattaacgagaaatttc
                  ************************************************************

Lep0813_Iran      tcttttattatattaaggtcttatattcaataatgatgtaaaattgcaaattttaaggag
Lep0816_Iran      tcttttattatattaaggtcttatattcaataatgatgtaaaattgcaaattttaaggag
Lep0941_Russia    tcttttattatattaaggtcttatattcaataatgatgtaaaattgcaaattttaaggag
Lep0944_Russia    tcttttattatattaaggtcttatattcaataatgatgtaaaattgcaaattttaaggag
Lep0963_Russia    tcttttattatattaaggtcttatattcaataatgatgtaaaattgcaaattttaaggag
                  ************************************************************

Lep0813_Iran      tatttaaattatactaaggcttaaagactatttctttataaataattttgaagattatta
Lep0816_Iran      tatttaaattatactaaggcttaaagactatttctttataaataattttgaagattatta
Lep0941_Russia    tatttaaattatactaaggcttaaagactatttctttataaataattttgaagattatta
Lep0944_Russia    tatttaaattatactaaggcttaaagactatttctttataaataattttgaagattatta
Lep0963_Russia    tatttaaattatactaaggcttaaagactatttctttataaataattttgaagattatta
                  ************************************************************

Lep0813_Iran      gtttaccttaacttaaaaccttaataaaaaaaaaaggttctaataattatactaaaaata
Lep0816_Iran      gtttaccttaacttaaaaccttaataaaaaaaaaaggttctaataattatactaaaaata
Lep0941_Russia    gtttaccttaacttaaaaccttaataaaaaaaaaaggttctaataattatactaaaaata
Lep0944_Russia    gtttaccttaacttaaaaccttaataaaaaaaaaaggttctaataattatactaaaaata
Lep0963_Russia    gtttaccttaacttaaaaccttaataaaaaaaaaaggttctaataattatactaaaaata
                  ************************************************************
```

```
                                           g
Lep0813_Iran      gaaaaaaatgaaaataaattaataaaagaaaatttattattttaataaaaactttaaat
Lep0816_Iran      gaaaaaaatgaaaataaattaataaaagaaaatttattattttaataaaaactttaaat
Lep0941_Russia    gaaaaaaatgaaaataaattaataaaagaaaatttattattttaataaaaactttaaat
Lep0944_Russia    gaaaaaaatgaaaataaattaataaaagaaaatttattattttaataaaaactttaaat
Lep0963_Russia    gaaaaaaatgaaaataaattaataaaagaaaatttattattttaataaaaactttaaat
                  ************************************************************

                                                           a
Lep0813_Iran      cattttatttttaaataattaaatataatagatgaataaatgattttaatataaaaaaat
Lep0816_Iran      cattttatttttaaataattaaatataatagatgaataaatgattttaatataaaaaaat
Lep0941_Russia    cattttatttttaaataattaaatataatagatgaataaatgattttaatataaaaaaat
Lep0944_Russia    cattttatttttaaataattaaatataatagatgaataaatgattttaatataaaaaaat
Lep0963_Russia    cattttatttttaaataattaaatataatagatgaataaatgattttaatataaaaaaat
                  ************************************************************

Lep0813_Iran      aaaataattaatcttgttaaaattataattatagttagtaagtaagattgattattaatt
Lep0816_Iran      aaaataattaatcttgttaaaattataattatagttagtaagtaagattgattattaatt
Lep0941_Russia    aaaataattaatcttgttaaaattataattatagttagtaagtaagattgattattaatt
Lep0944_Russia    aaaataattaatcttgttaaaattataattatagttagtaagtaagattgattattaatt
Lep0963_Russia    aaaataattaatcttgttaaaattataattatagttagtaagtaagattgattattaatt
                  ************************************************************

Lep0813_Iran      aaaaaattaataataattcatttagggaaaaatccaataaagggggtaatcctcctaat
Lep0816_Iran      aaaaaattaataataattcatttagggaaaaatccaataaagggggtaatcctcctaat
Lep0941_Russia    aaaaaattaataataattcatttagggaaaaatccaataaagggggtaatcctcctaat
Lep0944_Russia    aaaaaattaataataattcatttagggaaaaatccaataaagggggtaatcctcctaat
Lep0963_Russia    aaaaaattaataataattcatttagggaaaaatccaataaagggggtaatcctcctaat
                  ************************************************************

                                                                            a
Lep0813_Iran      gaaagaaaattaattaatatattgatttttataattggattgatattattaatgaatagt
Lep0816_Iran      gaaagaaaattaattaatatattgatttttataattggattgatattattaatgaatagt
Lep0941_Russia    gaaagaaaattaattaatatattgatttttataattggattgatattattaatgaatagt
Lep0944_Russia    gaaagaaaattaattaatatattgatttttataattggattgatattattaatgaatagt
Lep0963_Russia    gaaagaaaattaattaatatattgatttttataattggattgatattattaatgaatagt
                  ************************************************************

Lep0813_Iran      tgtttaatataaaataaatttattatattaaataatcaacatataattctaattataaat
Lep0816_Iran      tgtttaatataaaataaatttattatattaaataatcaacatataattctaattataaat
Lep0941_Russia    tgtttaatataaaataaatttattatattaaataatcaacatataattctaattataaat
Lep0944_Russia    tgtttaatataaaataaatttattatattaaataatcaacatataattctaattataaat
Lep0963_Russia    tgtttaatataaaataaatttattatattaaataatcaacatataattctaattataaat
                  ************************************************************

Lep0813_Iran      gaataaacgattaagaaaaaaattcataaattttctctaattataatggaagttaatatt
Lep0816_Iran      gaataaacgattaagaaaaaaattcataaattttctctaattataatggaagttaatatt
Lep0941_Russia    gaataaacgattaagaaaaaaattcataaattttctctaattataatggaagttaatatt
Lep0944_Russia    gaataaacgattaagaaaaaaattcataaattttctctaattataatggaagttaatatt
Lep0963_Russia    gaataaacgattaagaaaaaaattcataaattttctctaattataatggaagttaatatt
                  ************************************************************

Lep0813_Iran      caacctaaattattaatggaagaaaaagctattaatttacgtaaagaggtttgatttaat
Lep0816_Iran      caacctaaattattaatggaagaaaaagctattaatttacgtaaagaggtttgatttaat
Lep0941_Russia    caacctaaattattaatggaagaaaaagctattaatttacgtaaagaggtttgatttaat
Lep0944_Russia    caacctaaattattaatggaagaaaaagctattaatttacgtaaagaggtttgatttaat
Lep0963_Russia    caacctaaattattaatggaagaaaaagctattaatttacgtaaagaggtttgatttaat
                  ************************************************************
```

```
Lep0813_Iran    cctcctaaagctccaatgattacatttattgagataattaaaataataaaatttttattt
Lep0816_Iran    cctcctaaagctccaatgattacatttattgagataattaaaataataaaatttttattt
Lep0941_Russia  cctcctaaagctccaatgattacatttattgagataattaaaataataaaatttttattt
Lep0944_Russia  cctcctaaagctccaatgattacatttattgagataattaaaataataaaatttttattt
Lep0963_Russia  cctcctaaagctccaatgattacatttattgagataattaaaataataaaatttttattt
                ************************************************************
```

B

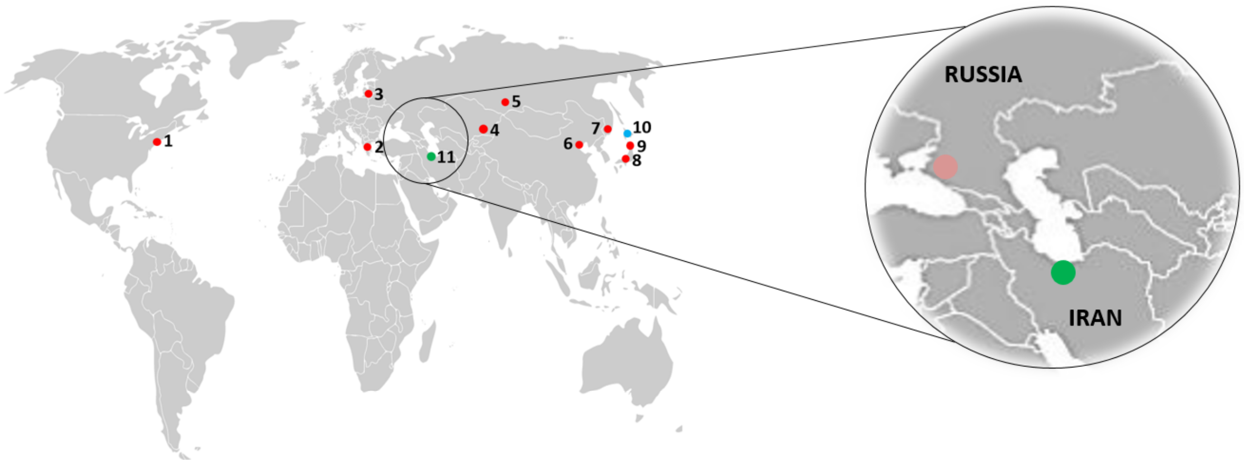

Name	Origin	Region	Supplier
Lep0813	Iran	Noor, Mazandaran	H. Rajaei
Lep0816	Iran	Noor, Mazandaran	H. Rajaei
Lep0941	Russia	Krasnodar Reg.	R.V. Yakovlev
Lep0944	Russia	Krasnodar Reg.	R.V. Yakovlev
Lep0963	Russia	Krasnodar Reg.	R.V. Yakovlev

Figure S2. A) Nucleotide alignment (MAFFT) of the mitochondrial *COI-ND2* region for five specimens from the Caucasus (Caspian Sea region), including L?_IR. The alignment shows that all five specimens appear to share the same mitochondrial haplotype. In red, sequence from Lep0816-Iran, corresponding to L?_IR. In blue, SNPs unique to the Caucasian specimens, relative to the other *Lymantria dispar* haplotypes (in grey). B) Map showing the sampling locations of the specimens. Background map is a cropped version of the one available at: https://commons.wikimedia.org/wiki/Maps_of_the_world#/media/File:BlankMap-World-v2.png (for information about license, follow the same link).
